# Novel applications of molecular imaging to guide breast cancer therapy

**DOI:** 10.1186/s40644-022-00468-0

**Published:** 2022-06-21

**Authors:** Christine E. Edmonds, Sophia R. O’Brien, David A. Mankoff, Austin R. Pantel

**Affiliations:** Department of Radiology, Hospital of the University if Pennsylvania, 3400 Spruce Street, Philadelphia, PA 19104 USA

**Keywords:** Molecular imaging, Breast cancer, PET biomarkers

## Abstract

The goals of precision oncology are to provide targeted drug therapy based on each individual’s specific tumor biology, and to enable the prediction and early assessment of treatment response to allow treatment modification when necessary. Thus, precision oncology aims to maximize treatment success while minimizing the side effects of inadequate or suboptimal therapies. Molecular imaging, through noninvasive assessment of clinically relevant tumor biomarkers across the entire disease burden, has the potential to revolutionize clinical oncology, including breast oncology. In this article, we review breast cancer positron emission tomography (PET) imaging biomarkers for providing early response assessment and predicting treatment outcomes. For 2-^18^fluoro-2-deoxy-D-glucose (FDG), a marker of cellular glucose metabolism that is well established for staging multiple types of malignancies including breast cancer, we highlight novel applications for early response assessment. We then review current and future applications of novel PET biomarkers for imaging the steroid receptors, including the estrogen and progesterone receptors, the HER2 receptor, cellular proliferation, and amino acid metabolism.

## Background

The role of molecular imaging, particularly positron emission tomography (PET), continues to increase and evolve in the evaluation of breast cancer. While anatomic imaging (including mammography, sonography, and MRI for diagnosis and assessment of disease extent within the breast, and computed tomography (CT) for systemic staging) represent the traditional modalities utilized in breast cancer evaluation, molecular imaging offers the unique advantage of providing in vivo biochemical or metabolic information specific to the tumor. Although molecular imaging includes a variety of imaging detector technologies and probes, this review focuses on PET imaging, which is the most rapidly evolving molecular imaging technology with a growing number of novel tracers.

PET provides quantitative images of biologic processes at acceptably low patient radiation doses. In addition, among the various molecular imaging technologies, PET offers high sensitivity and relatively better spatial resolution, improving detection and quantification of regional tracer uptake. It also permits considerable flexibility in imaging probe design that enables the ability to target a wide variety of molecular processes of importance to cancer. The rapid translation of PET to clinical oncology in the late 1990’s and early 2000’s was due to advances in imaging technology, including combined PET/CT and widespread commercial production of the glucose analog FDG. Although utilization and potential applications of FDG-PET/CT continue to grow, novel PET tracers also demonstrate great potential to transform clinical breast oncology.

New oncologic therapies that target specific biologic pathways continue to transform clinical breast oncology and improve prognoses. These targeted therapies are typically paired with diagnostic tests (companion diagnostics) that can predict and/or monitor treatment response, allowing for tumor-specific treatment optimization, deemed precision medicine. Thus, in addition to the development of new targeted therapies, research efforts have also sought to identify biological markers (or biomarkers) that can identify and quantify the biologic pathway that is targeted for therapy, and thus predict drug efficacy and quantify a pharmacologic effect.

Traditional oncologic biomarkers are tissue-based assays. For example, immunohistochemistry (IHC) for expression of various cell receptors (e.g. the estrogen receptor (ER)) has become standard of care in the diagnosis and treatment of breast cancer. However, there are inherent limitations to tissue-based bioassays. The invasiveness of the biopsies limits the frequency and potential sites of sampling, as well as the number of disease sites that can be sampled. With limited sites of disease sampled, particularly in the metastatic setting, heterogeneity of the tumor burden cannot be assessed or incorporated into the treatment plan. Therefore, imaging biomarkers are of great interest to overcome these limitations by offering a non-invasive assessment of the full burden of disease [1]. PET biomarkers for breast cancer not only assess biologically-relevant markers, but also provide means of assessing response early in the course of drug treatment, often before anatomic changes are present on conventional imaging, and also predict long-term response. In the following review, we discuss novel applications of FDG-PET/CT for early disease assessment and highlight the most promising novel PET tracers beyond FDG that are poised to impact breast oncology. The biologic pathways and their respective molecular imaging probes covered in this review are included in the Table 1.

**Table 1:** Summary of the biologic pathways and their respective molecular imaging probes included in this review

**Biochemical/Metabolic Target**	**Radiotracer/s**
**Glucose metabolism**	^18^F-fluorodeoxyglucose (FDG)
**Steroid Receptor Expression**	
Estrogen Receptor	^18^F-fluoroestradiol (FES)
Progesterone Receptor	^18^F-fluoronorpregnenedione (FFNP)
Androgen Receptor	^18^F-fluorodihydrotestosterone (FDHT)
**HER2 Receptor Expression**	^89^Z-trastuzumab, ^64^Cu-DOTA-trastuzumab,^18^F-fluorobenzamidoethylmaleimide (FBEM)
**Cell proliferation: Thymidine analog**	^18^F-fluorothymidine (FLT)
**Amino Acid transport and metabolism**	^18^F-flurocyclobutane-1-carboxylic acid (^18F^-fluciclovine)^18^F-fluoroglutamine (^18^F-Gln)
**Poly (ADP-ribose) polymerase-1 (PARP-1) expression**	^18^F-FluorThanatrace

### Glucose metabolism imaging

FDG is the most studied and clinically utilized radiotracer in oncologic molecular imaging. FDG regional tumor uptake imaged after injection (typically 1 h) reflects regional glucose metabolic flux through the rate-limiting enzyme, *hexokinase* [2, 3]*.* The utility of FDG-PET across many types of malignancies highlights the ubiquity of dysregulated glucose metabolism in most cancers. The regional retention of FDG at delayed times following injection, typically at 1 h, reflects the rate of glycolysis. FDG-PET/CT is now an established modality for staging and restaging many types of malignancy, including breast cancer [2–4]. The National Comprehensive Cancer Network recommends consideration of FDG-PET/CT in the workup of Stage III and IV breast cancers, for staging and restaging [5]. FDG-PET/CT is highly sensitive for the detection of extra-axillary nodal as well as distant metastases [6–8], and is among the most accurate imaging modalities for staging recurrent breast cancer, with high sensitivity and specificity for disease beyond the breast [9–11]. FDG-PET/CT offers high accuracy, as compared to conventional anatomic imaging and bone scan, for detection of bone metastases, particularly lytic lesions, as it images the tumor activity itself rather than osseous reaction to tumor [12–14].

The role of FDG-PET/CT continues to evolve beyond staging/restaging applications. Multiple early studies demonstrated that FDG avidity is a prognostic marker for breast cancer; higher baseline tumor uptake is associated with worse clinical outcomes [15–19]. In addition, because glucose metabolism is downstream of numerous therapeutic targets, and because changes in tumor metabolism of glucose occur earlier than anatomic changes in tumor size, FDG-PET/CT offers emerging applications in the early assessments of treatment response. FDG offers particular value in the setting of neoadjuvant therapy, where patients with large locally advanced breast cancers are treated with drug therapy to preoperatively reduce the tumor volume and thus enable breast conservation therapy. FDG-PET/CT offers high sensitivity as an early predictor of response to neoadjuvant therapy [10, 20–25]. Early prospective clinical trials of patients with Stage II or III breast cancer (trials included patients with multiple histologic subtypes, on any neoadjuvant therapy regimen) demonstrated that relative declines in tumor standardized uptake value (SUV) of 45–50% after the first cycle of neoadjuvant chemotherapy accurately predicted pathologic response [26, 27]. A meta-analysis of 745 breast cancer patients on various neoadjuvant regimens also found that FDG-PET/CT serves as an early predictor of treatment response, with a sensitivity and specificity of 81 and 79%, respectively, and with a trend toward higher sensitivity after the second treatment cycle as compared to the first [22].

Although these early studies together suggest a valuable role for FDG-PET/CT as an early predictor of treatment response, it is not yet widely utilized in this role. This could be due to a lack of robust data to guide utilization of FDG-PET/CT to alter treatment course, and lack of evidence supporting improved outcomes [23, 28, 29]. Furthermore, the majority of the early trials investigating FDG-PET/CT for predicting response to neoadjuvant therapy were conducted on populations comprised of multiple subtypes of breast cancer. Because baseline FDG uptake and kinetics, as well as changes in uptake on treatment, vary by histologic tumor subtype [12, 30–32], the heterogeneity of the study populations limit the ability to define baseline FDG uptake values and change thresholds that accurately predict or define early response by subtype [12, 29]. Thus subtype-specific trials are needed to direct clinical FGD-PET/CT assessment of early neoadjuvant response. Several early subtype-specific trials, including for triple negative and human epidermal growth factor 2 (HER2)-postive breast canci, have been conducted to date.

Triple negative breast cancer (TNBC), defined by lack of expression of the ER, progesterone receptor (PgR), and HER2, accounts for 10–20% of invasive breast cancer, and carries a relatively worse prognosis, with higher rates of metastasis and recurrence, and higher mortality [33, 34]. This cancer subtype lacks targeted treatment options, and is frequently resistant to cytotoxic chemotherapy [35, 36]. Thus, imaging-based assessments of early neoadjuvant response is of great interest for TNBC. Consistent with its inherent clinical aggressiveness, TNBC demonstrates high baseline FDG uptake compared to other subtypes [16, 37], suggesting potential for FDG-PET as a potential biomarker of early treatment response. A study of 50 subjects with stage II or III TNBC demonstrated that the change in SUV between the baseline and follow-up study, performed after the second cycle of neoadjuvant therapy, was more predictive of pathologic outcome compared to the absolute value of the maxiumum SUV (SUV_max_) on either study. SUV_max_ decreased by a mean of 72% in patients who achieved pathologic complete response (PCR) compared to 38% in patients who did not, and a cutoff of 50% decrease in SUV_max_ provided the highest accuracy (80%) for predicting PCR, with a positive predictive value (PPV) of 67% and a negative predictive value (NPV) of 96%. However, while a cutoff of 42% offered a slightly lower accuracy of 74% (PPV 59%, NPV 100%), it had superior accuracy for predicting relapse, and thus was selected as the cutoff to define metabolic response [38]. This study confirmed results of a similar, smaller study of TNBC patients by the same team of investigators, which also demonstrated that, after screening for various cutoffs, a cutoff of 42% decrease in SUV_max_ from the baseline FDG-PET/CT to the follow-up study (performed after the second cycle of neoadjuvant therapy) best predicted disease-free survival [39]. The results from these investigations suggest very high specificity of FDG-PET/CT, thus indicating that the likelihood that FDG-PET/CT would fail to recognize a good neoadjuvant response in triple negative breast cancer patients is extremely unlikely [38].

Another study of mixed tumor subtypes also demonstrated that decrease in FDG uptake on the follow-up scan (performed 6-8 weeks following treatment initiation) compared to the baseline scan was predictive of PCR in the cohort of TNBC subjects [40]. However, yet another trial of mixed tumor subtypes did not demonstrate that decreased FDG uptake on follow-up was predictive of PCR in the TNBC cohort [37]. The discrepant results between these various studies could be related to variability among the studies, such as heterogeneous neoadjuvant regimens, timing between baseline and follow-up on FDG-PET scans, or small study numbers. Thus, larger clinical trials are warranted to better understand the potential role of FDG-PET/CT for the prediction and early assessment of neoadjuvant therapy for TNBC. In addition, despite early studies that have defined specific FDG-PET criteria for predicting neoadjuvant response in this patient population, FDG-PET/CT cannot distinguish a partial response from a complete response as accurately as pathology can. Because the standard of care is to proceed with surgery, either lumpectomy or mastectomy, following neoadjuvant therapy, pathology, the gold standard, is readily obtained, limiting the current role for FDG-PET/CT in the neodajuvant setting. However, as previously discussed by Ulaner, the role for FDG-PET/CT could expand if future studies define guidelines to alter neoadjuvant therapy regimens early in the course of therapy, similar to the current strategy for lymphoma [41].

FDG-PET/CT also offers a means of early assessment for HER2-targeted neoadjuvant therapy. Multiple studies have demonstrated marked and rapid declines in FDG uptake in HER2-positive breast cancer following initiation of HER2-positive neoadjuvant therapy [23, 29, 42]. A prospective trial of HER2-breast cancer subjects treated with the anti-HER2 drug trastuzumab plus taxane-based neoadjuvant therapy showed that low residual uptake, defined as SUV_max_ < 2.1, following the first neoadjuvant cycle predicted PCR [43]. A similar study of FDG-PET following the second treatment cycle confirmed that low post-treatment SUV_max_ was predictive of PCR, with an accuracy of 90% [44]. In the Neoadjuvant Lapatinib and/or Trastuzumab Treatment Optimization (NeoALTTO) trial, among the subset of patients who underwent FDG-PET/CT ad baseline and again at 2-6 weeks following neoadjuvant initiation, metabolic response was already detectable in primary tumors at 2 weeks, and was highly correlated with metabolic responses at 6 weeks. Furthermore, PCR was associated with greater declines in FDG uptake at both two and six weeks [42].

The multicenter phase II AVATAXHER trial investigated the use of FDG-PET for guiding neoadjuvant therapy in HER2-postive breast cancer patients. The trial assessed FDG-PET for predicting PCR early in the course of neoadjuvant docetaxel plus trastuzumab, and to determine if the addition of the angiogeneisis inhibitor bevacizumab could improve response among patients deemed unlikely to respond to the initial treatment regimen. Sixty-nine of the enrolled 142 subjects were predicted to be responders to the standard neoadjuvant regimen based on change in FDG uptake following the first cycle of therapy, and 37 of the 69 (53.6%) demonstrated PCR. The 73 subjects assessed as non-responders based on FDG-PET were randomized to continue the standard therapy versus to receive bevacizumab in addition to the standard regimen. Twenty-one of 48 (43.8%) of the non-responders who received the addition of bevacizumab demonstrated PCR, compared to just six of 25 (24%) of the non-responders who did not receive bevacizumab [45]. This trial offers a preliminary look at the value of FDG-PET for guiding or altering neoadjuvant therapy early in the course of treatment, to minimize treatment failure and optimize chances of PCR.

More recently, studies have investigated the use of FGD-PET/CT to identify HER2-positive breast cancer patients who warrant neoadjuvant therapy intensification, versus those who warrant treatment de-escalation. The TBCRC026 phase II study of stage II and III ER-negative, HER2-positive patients sought to identify early measurements of SUV_max_ corrected for lean body mass (SUL_max_) to predict PCR on patients treated with neoadjuvant pertuzuab and trastuzumab. Patients underwent FDG-PET/CT as baseline and again 15 days after initiating neoadjuvant therapy. Although the primary objective (to test the null hypothesis that the area under the curve of percent change in SUL_max_ between the two scans predicting PCR is </= 0.65) was not met, there was a significant difference in median percent reduction in SUL_max_ between those who achieved PCR and those who did not, suggesting that once optimized, quantitative FGD-PET/CT measures may facilitate tailoring therapy [46]. Another phase II trial of HER-positive breast cancer patients sought to assess early metabolic to neoadjuvant therapy (randomized to receive trastuzumab plus pertuzumab versus trastuzumab, pertuzumab, docetaxel, and carboplatin), in patients with Stage I-IIIA breast cancer with FDG-PET, as well as the possibility of chemotherapy de-escalation based on pathologic response-adapted strategy. The results showed that FDG-PET identified patients who were likely to benefit from dual-therapy HER2 blockade with trastuzumab and pertuzumab, without chemotherapy. Although the final results of this study, including results for the three-year invasive disease-free survival endpoint, are pending, the study suggests that FGD-PET (or PET-CT) may offer a valuable imaging bioassay to select patients who do not warrant chemotherapy [47].

**Fig. 1:**
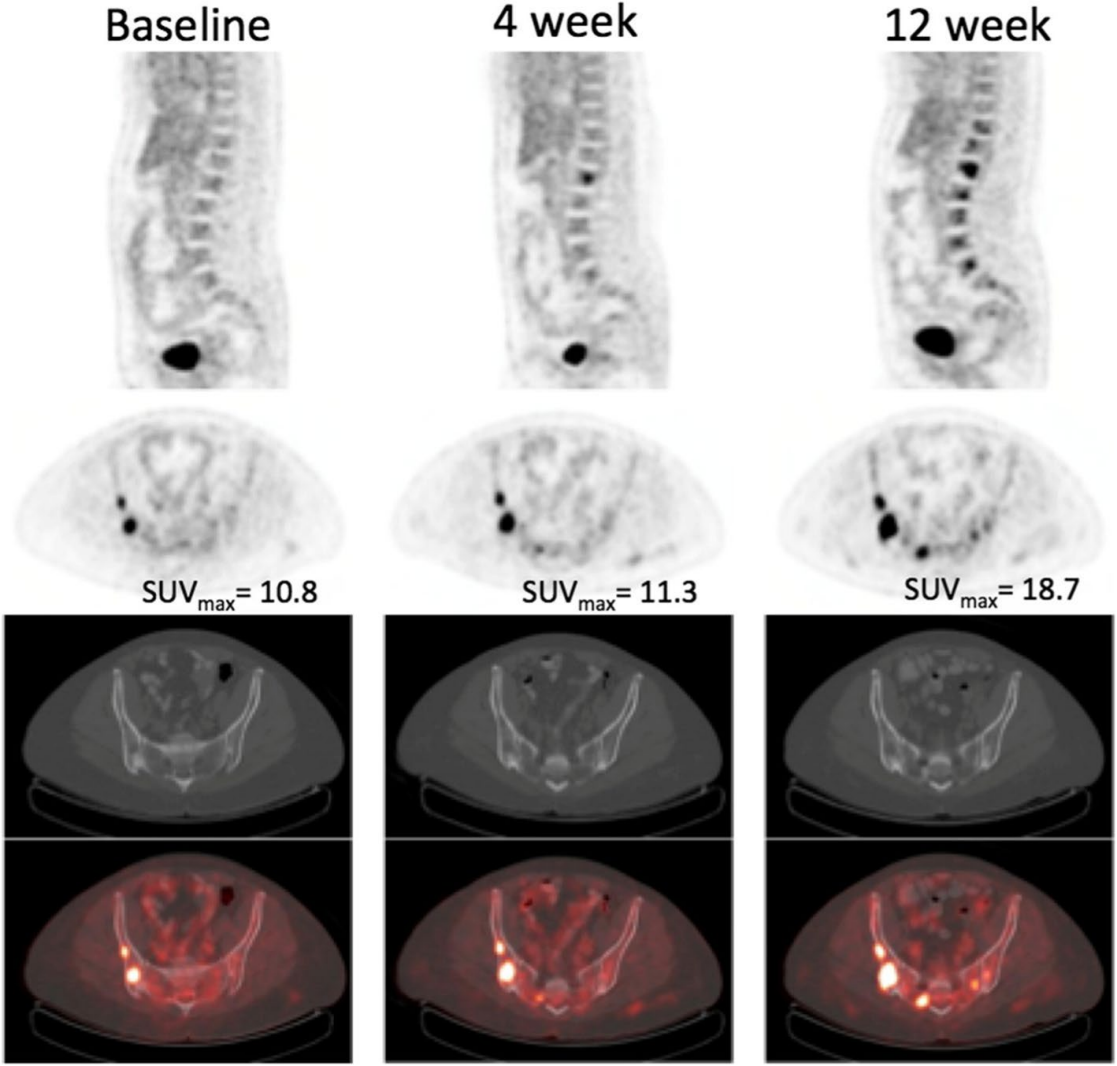
This patient with ER-positive breast cancer started a new endocrine therapy. Her FDG-avid osseous lesions did not demonstrate a decrease in FDG uptake to indicate a metabolic response at 4 weeks and 12 weeks. The patient progressed relatively quickly, in just over 100 days

FDG-PET/CT also offers value as an early assessment of treatment of metastatic (Stage IV) disease. While anatomic imaging, usually CT, has traditionally been the primary means of measuring treatment response, metabolic tumor changes on FDG-PET/CT likely offer a better, and earlier, response assessment compared to CT [48]. Early studies of FDG-PET demonstrated that it could distinguish response from non-response among patients with metastatic breast cancer, including in patients with bone-dominant disease, early in the course (following 1-3 cycles) of therapy [49–52]. Figure 1 demonstrates how FGD/CT can be utilized to demonstrate non-response early in the course of therapy: a patient with ER-positive metastatic breast cancer began a new endocrine therapy. Her FDG-avid osseous lesions did not demonstrate decreased FDG uptake to indicate metabolic response at four weeks or at 12 weeks. She ultimately had rapid disease progression, in just over 100 days. Subsequent studies investigated the utility of FDG-PET/CT to distinguish response from nonresponse among specific tumor subtypes on defined treatment regimens [53–55]. For example, a phase Ib study of metastatic breast cancer patients treated with the class I phospoinositide-3-kinase inhibitor buparlisib and the aromatase inhibitor letrozole demonstrated that a lack of metabolic response on FDG-PET/CT performed 2 weeks post treatment initiation compared to the pretreatment study was associated with rapid disease progression. In addition, there was a correlation between the decrease in FDG uptake relative to baseline and the duration of treatment, suggestive that the decreased tumor metabolism was predictive of treatment response [54]. A similar phase II study of HER2-positive metastatic breast cancer patients explored the predictive value of FDG-PET/CT performed at baseline, week one, and week eight of treatment with the anti-HER2 monoclonal antibody trastuzumab in combination with the tyrosine kinase inhibitor lapatinib. A lack of metabolic response at 1 week compared to baseline was associated with a lack of response by RECIST, with a negative predictive value of 91%, suggesting that very early FDG-PET/CT may allow for the selection of patients who should be treated with targeted therapy, while minimizing chemotoxicity among patients who are unlikely to benefit [53].

FDG-PET/CT has also shown utility in assessing early response to ER-targeted therapy. Pioneering work by Dehdashti, Mortimer and colleagues showed that the agonist “flare” of early tamoxifen treatment is associated with an increase in FDG uptake and predicts response to therapy [56]. Later worked showed that the increase in FDG uptake in response to estradiol infusion predicted both response and long-term outcome [57]. Similarly, studies have shown the reduction in estrogen agonists accompanying treatment with aromatase inhibitors results in a lowering of FDG uptake in both primary breast cancer and metastatic disease [58, 59].

Finally, research suggests that FDG-PET/CT may be particularly beneficial in the early assessment of osseous breast cancer metastases to therapy. While bone is among the most common sites of breast cancer metastases, traditional imaging modalities, including CT, MRI, and bone scan, suffer from limited accuracy for assessment of osseous metastases to systemic therapy [18, 48, 60]. Thus, use of FDG-PET/CT for this application continues to grow. A preliminary retrospective study by Specht et al. of 28 patients with bone dominant metastatic breast cancer found that higher baseline FDG uptake in skeletal metastases predicted a shorter time to the first skeletal-related event, such as pathologic fracture or spinal cord compression [51]. In addition, percent decrease in FDG uptake as measured by SUV was predictive of time to disease progression [51].

There is limited research to date comparing the accuracy of conventional imaging to FDG-PET/CT for early assessment and prediction of treatment efficacy in patients with metastatic breast cancer. However, a retrospective study by Riedl et al. included 65 patients with metastatic breast cancer who underwent both FDG-PET/CT and contrast enhanced CT at baseline and again within 90 days (mean interval from start of treatment to follow-up imaging was 55 days) of initiating systemic therapy. The results demonstrated that FDG-PET/CT more accurately predicted both progression-free and disease-specific survival compared to the standard Response Evaluation Criteria in Solid Tumors CT evaluation [61]. Furthermore, the differences in response assessment between CT and FDG-PET/CT were driven by the differences in patients with osseous metastases [61]. These results suggest a promising role for FDG-PET/CT for early treatment assessment and response prediction in two primary ways: (1) avoiding the discontinuation of treatment regimens among patients who are having a response by FDG-PET/CT that cannot be captured on CT and appear to falsely represent progression of disease, and (2) allowing early changes in therapy among patients who appear to have stable disease on CT, but who show no treatment response by FDG-PET/CT [61]. However, FGD-PET/CT is not yet widely clinically utilized for this purpose, and larger studies are warranted to demonstrate clinical value.

### Steroid receptor expression imaging

#### Estrogen receptor

Determination of hormone receptor status, both the ER and the PgR, in breast cancer is standard of care, as the receptor status has both prognostic implications and dictates therapy. The United States National Comprehensive Cancer Network (NCCN) recommends estrogen receptor testing in all primary and metastatic breast cancers, approximately 70–80% of which are ER-positive [62, 63]. The most common method of assessing and quantifying ER expression is IHC of biopsy specimens. However, an imaging assay of ER expression offers several potential advantages over IHC, including noninvasiveness, ability to measure ER status across the entire disease burden rather than at the single biopsy site, and potential for serial evaluation. Biopsy of metastatic sites is often technically challenging, is associated with higher morbidity compared to biopsy of the primary breast tumor, and is less accurate, particularly in the case of osseous metastases. Furthermore, ER expression is frequently heterogeneous, with expression in the primary tumor not necessarily predicting similar ER status across all of the sites of metastases [64–66]. An imaging assay thus offers the potential to simultaneously assess ER expression at all disease sites, and optimally select patients for endocrine therapy and monitor early response.

PET tracers for imaging the ER are analogs of estradiol. Estradiol is the strongest estrogen, and binds with high affinity to the ER in cell nuclei throughout the body. 16α-^18^F-fluoro-17β-estradiol (FES), first synthesized in the 1980s, binds to both the ER and to sex-hormone-binding globulin (important for transportation in the bloodstream of both FES and estradiol) with similar affinity to estradiol. Over the past three decades, numerous studies of FES-PET in breast cancer have demonstrated its accuracy as a biomarker of functional ER-expression, and have shown its value in predicting and assessing early response to endocrine therapy. In 2016, FES, under the trade name EstroTep®, was approved for clinical use in France in patients with recurrent, initially ER-positive breast cancer in whom biopsy is deemed impossible [67, 68]. In 2020, FES, under the trade name Cerianna®, was approved by the U.S. Food and Drug Administration for use as an adjunct to biopsy in patients with recurrent or metastatic ER-positive breast cancer [68].

ER-targeted therapies have led to markedly improved outcomes in patients with ER-positive disease, with a 5-year overall survival rate of 91–94% compared to 77–84% in patients with ER-negative breast cancer [63]. FES-PET/CT provides a non-invasive, whole body assessment of functional ER-expression which has been validated as a marker of ER expression by both radioligand binding assay and IHC performed on tissue samples [69, 70]. FES-PET/CT thus evaluates the entire burden of ER-positive disease, assesses response to therapy, and identifies receptor heterogeneity among sites of disease. With serial imaging, it identifies changes in receptor status over time, noting that many of these scenarios remain research indications in the United States if performed without confirmatory tissue sampling.

**Fig. 2:**
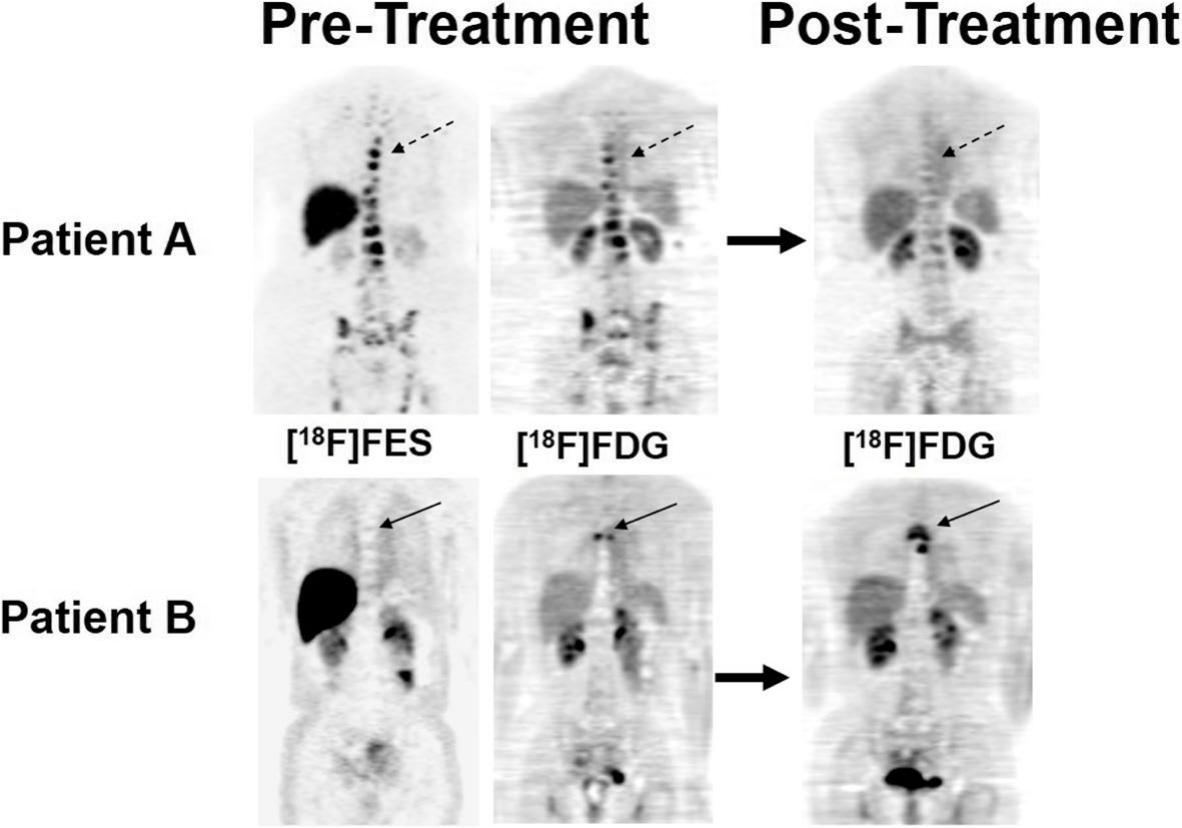
Pre-treatment FES-PET (far left) and FDG-PET (mid) scans are shown, with post-treatment FDG-PET follow-up (right). Patient A (top images) revealed breast cancer metastasis to bone (multi-level vertebral and pelvic metastases, representative lesions indicated by dashed arrow and arrowhead, respectively) which demonstrated homogenous FES- and FDG-positivity. FDG imaging 3 months following hormonal therapy depicts extensive response to therapy, as predicted by homogenously FES-positive disease. Patient B (bottom images) revealed breast cancer metastasis to bone (upper thoracic vertebra, narrow arrow) which demonstrated FES-negativity and FDG-positivity. FDG imaging following 6 months of hormonal therapy depicts progression, as predicted by FES-negative disease. Figure adapted with permission from Linden et al. J Clin Oncol 2006 [65]

The normal biodistribution of FES includes highly ER-expressing organs (e.g. uterus) as well as organs involved in its metabolism and excretion such as the liver, biliary system, bowel (through enterohepatic circulation), kidneys, ureters, and bladder [71, 72]. Comparison of FES-PET to tissue assay of ER and response to therapy suggest that FES uptake with SUV >/= 1.5 is roughly equivalent to ER-positivity by tissue assay. When sites of known metastatic disease are considered FES positive (with SUV >/= 1.5 or qualitatively clearly above local background), disease was more likely to respond to ER-targeted therapy, and FES-negative disease was strongly correlated with poor response to ER- therapy [73, 74]. For example, Linden et al. found that 46% (11 of 24) of patients with FES-positive disease responded to ER-therapy, while 0% (0 of 15) of patients with FES-negative disease responded [65]. In a 2014 phase II study, Peterson et al. found that 64% (9 of 14) of patients with FES-positive disease responded to ER-therapy, and again demonstrated that patients with FES-negative disease had minimal or no response to endocrine therapy (five of six demonstrated progression of disease, and one had stable disease at 6 months) [75]. Figure 2 illustrates how individual patients’ baseline FES-PET scan can predict response to endocrine therapy: the patient with FES-positive disease demonstrated robust response to endocrine therapy, while the patient with FES-negative disease progressed on endocrine therapy [65].

In addition to the association between FES-positivity with improved clinical outcomes on ER-therapy, quantitatively higher FES uptake may also predict disease response, and can affect initial management decisions. In 2001, Mortimer et al. demonstrated that among advanced breast cancer patients treated with tamoxifen, those with clinical response to therapy had higher baseline FES uptake (SUV, 4.3 +/− 2.4) than did non-responders (SUV, 1.8 +/− 1.3) [56]. Higher FES uptake on baseline imaging has also been used to identify patients with aromatase-resistant disease who would benefit from simultaneous therapy with both a histone deacetylase inhibitor and aromatase inhibitor [76]. Additionally, Kurland et al. found that, among patients with highly FDG-avid lesions, those patients whose lesions also demonstrated high FES uptake had longer median progression-free survival than patients whose lesions had low ^18^F-FES uptake [77].

Prior studies demonstrate that FES-PET/CT can accurately characterize disease heterogeneity both spatially (among different disease sites at one point in time) and temporally (the same sites of disease on serial scans). The qualitative assessment of FES uptake in metastatic breast cancer sites is associated with patient outcomes. In 2020, Boers et al. found that among metastatic breast cancer patients treated with combined endocrine therapy and a cyclin-dependent kinase inhibitor, patients with homogenous FES-positive disease had longer median time to disease progression (73 weeks) compared to patients with heterogeneous FES uptake (27 weeks) or FES-negative disease (15 weeks) [78]. Identifying patients with spatially heterogeneous disease enables clinicians and patients to expect the possibility of decreased response to ER-therapy and to explore alternative treatment regimens early in the course of treatment, when necessary. Identifying changes in ER-expression over time can also inform treatment decisions. Currin et al. reported a case in which serial FES-PET/CTs identified the initial loss of ER-expression in a patient who progressed on both ER therapy and then chemotherapy, with later return of ER expression and subsequent successful salvage ER therapy [79].

Finally, FES-PET/CT has also been used to evaluate ER blockade by different endocrine therapies. In 2011, Linden et al. demonstrated that estrogen receptor blocking agents (tamoxifen and fulvestrant) decreased FES tumor uptake to a greater extent compared to estrogen concentration lowering agents (aromatase inhibitors) [80]. Linden et al. also found that 100% (5 of 5) of patients treated with tamoxifen demonstrated complete ER blockade, while only 26% (4 of 11) of patients treated with fulvestrant demonstrated complete blockade [80]. A 2015 study by van Kruchten et al. demonstrated that incomplete tumor blockade as assessed by FES-PET/CT was associated with early disease progression [81]. FES-PET/CT’s ability to evaluate ER blockade was utilized in a 2017 phase I dose escalation trial of a new selective estrogen receptor degrader to identify the optimal dosing in patients with ER-positive metastatic breast cancer [82]. These early studies highlight the value of FES-PET/CT to both guide dosing of established endocrine therapies, as well as to provide a tool to test and guide dosing of endocrine therapy agents and combination regimens.

#### Progesterone receptor

Approximately two thirds of primary breast cancers express the PgR, and the majority of these are also ER-positive [83]. The biology of these two receptors is linked: the ER regulates the synthesis of the PgR, and expression of the PgR indicates a functional ER pathway [84–86]. PgR expression is independently associated with both disease-free and overall survival, and among ER-positive breast cancers, those that are also PgR-positive are more likely to respond to endocrine therapy compared to those that are PR-negative [87]. Thus, PgR status, like ER status, of breast cancer biopsy specimens is routinely assessed at diagnosis, typically by IHC, and is useful to guide therapy and inform prognosis.

Expression of the PgR, as measured by PET, has been leveraged as an indicator of a functional estrogen receptor [88]. The most promising and widely studied PgR radioligand to date is 21-^18^F-fl uoro-16α,17α -[(R)-(1′ -α -furylmethylidene)dioxy]-19-norpregn-4-ene-3,20-dione (FFNP), with high affinity and selectivity for the PR [89]. The first-in-human studies of the FFNP demonstrated greater tumor-to-normal uptake ratios of FFNP in PR-positive tumors compared to PR-negative tumors [88]. More recently, Dehdashti et al. conducted a phase II trial in 43 women with postmenopausal advanced ER-positive breast cancer, to explore the hypothesis that a brief estradiol challenge would increase tumor expression of the PgR only in patients with a functional ER. FFNP-PET/CT was performed before and after a one-day estradiol challenge. The results showed a post-challenge increase in tumor FFNP uptake only in the 28 subjects who had clinical benefit (responders) from endocrine therapy, but not among the 15 non-responders, thus indicating perfect sensitivity and specificity. The study also demonstrated significantly longer survival in the responding subjects [90]. This study suggests that FFNP-PET/CT may be a valuable predictor of response to endocrine therapy among ER-positive breast cancer patients, by differentiating those cases with functional ERs from those without, and thus appropriately guiding therapy.

#### Androgen receptor

In addition to imaging the ER and PR, a recent study demonstrated the feasibility and potential utility of imaging the androgen receptor (AR) in breast cancer, using the agent ^18^F-16β-^18^F-fluoro-5α-dihydrotestosterone [91], an agent that has been more widely tested in prostate cancer [92]. Further studies are needed to define the potential clinical utility of this tracer for breast cancer.

### HER2 receptor imaging

HER2 is a tyrosine kinase receptor protein, belonging to the epidermal growth factor (EGF) family, and is among the strongest prognostic biomarkers for breast cancer [93]. Breast cancers with HER2 protein overexpression (defined as 3+ immunohistochemistry status) or an elevated HER2 gene copy (six or more as evaluated by in situ hybridization) are considered clinically HER2-positive, and comprise 15–30% of invasive breast cancers. HER2-positivity is associated with a relatively aggressive course and poor prognosis, with higher risk of both relapse and deat [94–97]. In addition, HER2 overexpression results in impaired response to both endocrine therapy [97] and cytotoxic chemotherapy [97, 98].

In recent years, HER2-targeted therapies, including the monoclonal antibodies trastuzumab and pertuzumab, the antibody-drug conjugate trastuzumab-emtansine, and the tyrosine kinase inhibitor lapatinib are approved for treatment of HER2 positive breast cancer [99]. Targeted HER2 therapy has greatly improved the survival of HER2-positive breast cancer patients [100, 101], and combination therapy has increased efficacy in the neoadjuvant setting [102, 103] and improved survival in the metastatic setting [104, 105]. However, both primary as well as acquired resistance to anti-HER2 therapies remain problematic, and limit the efficacy of current regimens [106–108]. In addition, HER2-targeted therapies are associated with side effects, including cardiotoxicity [109, 110]. Thus, there is strong clinical need to better predict response to, and tailor, HER-targeted therapy, while minimizing treatment failure and corresponding unnecessary side effects.

Given the prognostic and treatment implications of HER2 expression, both the American Society of Clinical Oncology and the College of American Pathologists recommend determination of HER2 status for all invasive breast cancers. HER2 status is routinely assessed by IHC or fluorescence in situ hybridization of tissue biopsy or surgical specimens. Despite the clinical impact of HER2 assays, reliance on biopsy for assessment has significant limitations, including the quality of the tissue specimen for analysis and disease heterogeneity and sampling error. HER2 expression is often heterogeneous, with intra- and intertumoral heterogeneity as assessed by IHC as high as 13 and 30%, respectively [111]. A study by Santelli et al. demonstrated a clinically significant discordance between HER2 expression of the primary breast tumor and metachronous recurrence or metastasis in 21.5% of cases [112]. In addition, certain anatomic sites of metastases, such as bone, have high morbidity associated with percutaneous biopsy. Bone, a frequent site of breast cancer metastases, is also plagued by limited assessment of receptor status, including HER2, by IHC [113]. An imaging assay of HER2 status offers a noninvasive assessment of the entire breast cancer disease burden and may be used for serial assessments over time, to predict and assess response to HER2-targeted therapy.

Multiple imaging agents have been investigated for noninvasive in vivo assessment of HER2 expression, and include agents labeled with single photon radionuclides for single photon emission computed tomography (SPECT) and others labeled with positron emitting radionuclides for PET. Subsequent paragraphs in this section highlight the most studied and promising HER2 PET radionuclides, most of which are based on immune recognition. Radiolabeled Anti-HER2 immune-based tracers include immunoglobulins (trastuzumab and pertuzumab), immunoglobulin fragments, and engineered scaffold proteins such as affibodies and albumin-binding domain derived affinity proteins (ADAPTS). To date, clinical experience is limited to pilot and phase I studies.

Radiolabeled monoclonal anti-HER2 antibodies are among the most studied and most promising probes for molecular imaging of HER2 expression. A primary limitation of antibody-based imaging is the relatively large size of antibodies, which results in slow clearance from the blood pool and other compartments, as well as low tumor penetration, resulting in the need for a delay of 4-6 days between tracer injection and imaging to obtain satisfactory tumor-to-blood ratios, and thus requires radionuclides with long half-lives, such as ^64^Cu or ^89^Zr. One of the promising HER2 PET tracers is the radiolabeled monoclonal antibody ^89^Z-trastuzumab, with a half-life of 78.4 hours, permitting imaging up to 7 days following injection, to maximize HER2-positive tumor visualization [114, 115]. The pilot imaging trial of ^89^Z-trastuzumab-PET included 14 patients with HER2-positive breast cancer, and demonstrated excellent tumor uptake of the tracer on delayed imaging (approximately 5 days post injection) and visualization of nearly all known metastases as well as several occult metastases [116]. A second study of ^89^Z-trastuzumab-PET in 12 patients with metastatic breast cancer confirmed that optimal imaging required at least 4 days between injection and imaging [117]. A study of ^89^Z-trastuzumab-PET in 20 patients, including some with lesions of interest that couldn’t be biopsied, demonstrated that this novel imaging approach altered treatment decisions in 40% of cases [118]. Early research also shows utility of ^89^Z-trastuzumab-PET in predicting and assessing early treatment response. In the multicenter ZEPHIR study, a prospective investigation of ^89^Z-trastuzumab-PET in 60 patients with metastatic breast cancer found that having mostly HER2-positive lesions by ^89^Z-trastuzumab-PET and early metabolic response to T-DM1 treatment (defined by response on ^89^Z-trastuzumab-PET after 1 cycle) was predictive of greater response after three treatment cycles as well as longer progression-free survival [119]. This study confirmed that both loss of HER2 expression and HER2 heterogeneity are common following systemic treatment of initially HER2-positive breast cancer [119]. Similar early studies have also investigated trastuzumab labeled with ^64^Cu. A pilot study of ^64^Cu-DOTA-trastuzumab-PET in six patients with primary or metastatic HER2-positive breast cancer demonstrated safety and feasibility of this agent, as well, including for evaluation of HER2-postive brain metastases [120].

Multiple research groups have also investigated affibody molecules for imaging HER2. The much smaller size of these proteins compared to antibodies allows more rapid blood clearance and higher tumor penetration, thus precluding the need for delayed imaging. Preclinical studies of affibodies for HER2 imaging have utilized a variety of isotopes. Promising preclinical studies of an ^18^F-labeled antibody, N-[2-(4-^18^F-fluorobenzamido)ethyl]maleimide (FBEM), demonstrated that tracer uptake on PET correlated with HER2 receptor expression by IHC in a xenograft mouse model. Furthermore, following treatment with 17-DMAG, an inhibitor of heat shock protein 90 that decreases HER2 expression, HER2 expression decreased as measured by ^18^F-FBEM-PET, as well as on pathology [121]. Early proof-of-principal pilot human trials of HER2-targeted affibodies have also been conducted with both SPECT and PET, with current efforts seeking to assess safety, and optimize tracer dose and timing of imaging [110].

As is the case for ER-targeted therapy, the combination of HER2 PET imaging to assess the target and other imaging such as FDG-PET/CT to measure early response may by particularly helpful in guiding HER2-targeted therapy. A nice example is seen in the results of the SEPHIR trial, where the combination of pre-therapy ^89^Z-trastuzumab-PET and serial FDG-PET/CT provided high positive and negative predictive value for response to HER2-targeted therapy [119].

### Imaging tumor proliferation with thymidine analogs

Increased cellular proliferation is a hallmark of malignancy, including breast cancer, and is clinically relevant to tumor behavior and growth [122]. Markers of tumor proliferation may be used in combination with tumor size, grade, nodal status, and receptor status as a breast cancer prognostic indicator, and may also guide treatment [123]. Therefore, markers of cell proliferation offer useful imaging biomarkers for predicting and assessing early treatment response. A variety of laboratory assays have been investigated to quantify cell proliferation on tissue pathology specimens, including S-phase fraction and mitotic index. However, the most utilized and validated laboratory assay of proliferation is IHC of the human protein Ki67, expressed in the nuclei of all dividing cells during G1, S, G2, and M phases, but absent during G0 [123, 124]. Thus Ki67 represents the total cellular proliferation, is correlated with other markers of proliferation [123], and is expressed at higher levels in higher grader cancers compared to those of lower grade [123, 125]. However, Ki67, like other tissue-based assays, has limitations that may be overcome by an imaging biomarker of proliferation, including sample variation and heterogeneity. A study of Ki67 expression demonstrated significant variation between core biopsy specimens and surgical specimens, without interim therapy [126]. An imaging biomarker could overcome this limitation, while also allowing a noninvasive measure of proliferation across the disease burden and over time.

The most studied and promising approach to imaging proliferation to date is to image the salvage pathway of incorporating thymidine into DNA. While early studies investigated ^11^C-thymidine-PET to image tumor cell proliferation and treatment response, the short half-life of ^11^C and its complex metabolism limited its clinical utility [127, 128]. The fluorinated thymidine analog ^18^F-3′-deoxy-3′-fluorothymidine (FLT) has overcome these limitations, providing a more practical approach and currently representing the primary proliferation imaging agent. Cellular uptake of FLT is dependent on the activity of thymidine-kinase-1 (TK-1) which is relatively overexpressed in proliferating, including malignant, cells, but is low or absent in quiescent cells [129, 130]. FLT uptake on PET in breast cancer correlates with Ki67 expression [129, 131].

Compared to FDG-PET, FLT demonstrates lower cellular uptake, and has high uptake in the liver and bone marrow [20, 129]. Although these features make it unsuited for staging purposes, it offers significant potential value as an early marker and predictor of breast cancer treatment response. Multiple early studies of FLT-PET in breast cancer showed early changes in FLT uptake following initiation of chemotherapy [132–135]. A small study of FLT-PET breast cancer patients by Pio et al. demonstrated that mean change in FLT uptake in both primary and metastatic breast cancer following the first course of chemotherapy compared to baseline was significantly correlated with late changes in tumor marker levels. In addition, the early change in FLT uptake predicted late changes in tumor size on CT [135]. A pilot study by Kenny et al. found that FLT-PET can show proliferation changes as early as 1 week following chemotherapy, with significant differences in tracer uptake between treatment responders and non-responders [134]. A study by Contractor et al. demonstrated that decreased FLT uptake on PET 2 weeks after the first or second dose of docetaxel predicts anatomical tumor response at mid-therapy (following 3 cycles) with high sensitivity [133], and a pilot study also led by Contractor showed that change in FLT uptake 14 days after the first dose of docetaxel is correlated with decreased circulating tumor cells [132].

While early studies of FLT in breast cancer showed promise, several more recent studies of FLT-PET in the neoadjuvant setting demonstrate conflicting results. A study of 20 women found that baseline uptake on FLT-PET/CT, as measured by SUV, was significantly related to Ki67 expression. However, neither baseline FLT uptake nor the change in uptake following 1 cycle of neoadjuvant therapy was predictive of treatment response [136]. A recent multicenter phase II study performed as part of the ACRIN 6688 trial also sought to assess whether early changes in FLT-PET/CT are predictive of PCR among patients with invasive ductal carcinoma on neoadjuvant therapy. Patients underwent FLT-PET/CT at baseline, after the first treatment cycle (first post-treatment), and after completing treatment. While FLT uptake on the final post-treatment scan correlated with Ki67 expression on surgical pathology, there was only marginal difference in SUV_max_ percent change from baseline to the first post-treatment scan between patients with and without PCR [137]. Most of these studies to date have been conducted on patient populations with heterogeneous tumor subtypes and on a variety of neoadjuvant therapy regimens, confounding analyses.

A recent study by Romine et al. investigated FLT-PET/CT for assessing early response to aromatase inhibitors in patients with ER-positive operable breast cancer. The study included a subset of women who underwent paired FLT-PET/CT scans prior to endocrine therapy and again pre-operatively, with tissue samples also obtained prior to treatment and at surgery. While the post therapy SUV_max_ was significantly associated with post-therapy Ki67 in surgical specimens, the percent change in FLT uptake did not correlate with changes in Ki67. Although these results suggest that FLT-PET/CT performed pre-therapy and following a short exposure to endocrine therapy, or at a single time point following initiation of therapy, could add value in guiding treatment, further studies are needed to understand how best to apply FLT-PET/CT.

Preclinical trials also suggest a role for FLT-PET/CT in evaluating early response to cyclin-dependent kinase 4/6 (CDK4/6) inhibitors. Ma et al. demonstrated that FLT-PET/CT could immediately and accurately monitor Palbociclib (a CDK4/6 inhibitor) response in a mouse model of triple negative breast cancer [138]. In a similar preclinical study by Elmi et al., the investigators studied the effects of CDK4/6 inhibition in combination with estrogen-blockade in ER-positive breast cancer cell lines via in vitro FLT assays and FLT-micro-PET of a mouse model. The results demonstrated that FLT is sensitive to immediate changes in S-phase, suggesting a potential role for FLT-PET/CT in the early prediction of long-term treatment response in patients on this treatment regimen [139]. These preclinical studies demonstrate that translation of FLT-PET/CT to human studies for early CDK4/6 response assessment is warranted. The addition of other proliferation imaging agents that also reflect the cell cycle beyond the S-phase, for example the sigma-2 imaging agent ^18^F-Iso-1 [140, 141], may add further predictive value for predicting response to CDK4/6 inhibitors [139].

### Imaging amino acid transport and metabolism

Dysregulated metabolism in malignancy extends beyond accelerated glucose metabolism, providing additional opportunities for PET imaging [142]. Accelerated amino acid metabolism has also been leveraged for PET imaging. The synthetic amino acid anti-1-amino-3-^18^F-flurocyclobutane-1-carboxylic acid (^18^F-fluciclovine) received FDA approval in 2016 for imaging men with biochemical recurrence of prostate cancer [143]. As a non-metabolized synthetic amino acid, ^18^F-fluciclovine utilizes the same transporters as native amino acids, namely those involved in glutamine transport, but is not metabolized [144]. Consequently, ^18^F-fluciclovine washes out over time, and images are obtained soon after injection to measure amino acid transport [143], a distinctly different protocol than imaging trapped FDG an hour after injection. ^18^F-fluciclovine-PET, though, is not specific for prostate cancer and has been studied as an investigational agent in other malignancies, including breast cancer.

As a measure of amino acid metabolism, ^18^F-fluciclovine-PET has been studied in imaging breast cancer. A 2016 study of 37 women with a new diagnosis of locally advanced breast cancer demonstrated the ability of ^18^F-fluciclovine-PET to image both invasive lobular carcinoma (ILC) and invasive ductal carcinoma (IDC). In a subset of patients who were also imaged with FDG, those with ILC had greater uptake of ^18^F-fluciclovine compared to FDG, while those with IDC had lower uptake relative to ^18^F-fluciclovine [145]. Similar observations were published contemporaneously in a study of 12 women with 17 breast lesions [146]. These different patterns of uptake reflect underlying biologic differences between subtypes, interrogated with probes targeting different biologic pathways. This finding also suggests potential for imaging ILC, a subtype of breast cancer with suboptimal imaging options. Of note, a more recent study from 2021 demonstrated no significant differences in kinetic or static ^18^F-fluciclovine kinetic parameters between ER-positive, HER2-positive, and TNBC subtypes of breast cancer [147]. Utilizing ^18^F-fluciclovine in breast cancer remains an active area of research.

**Fig. 3:**
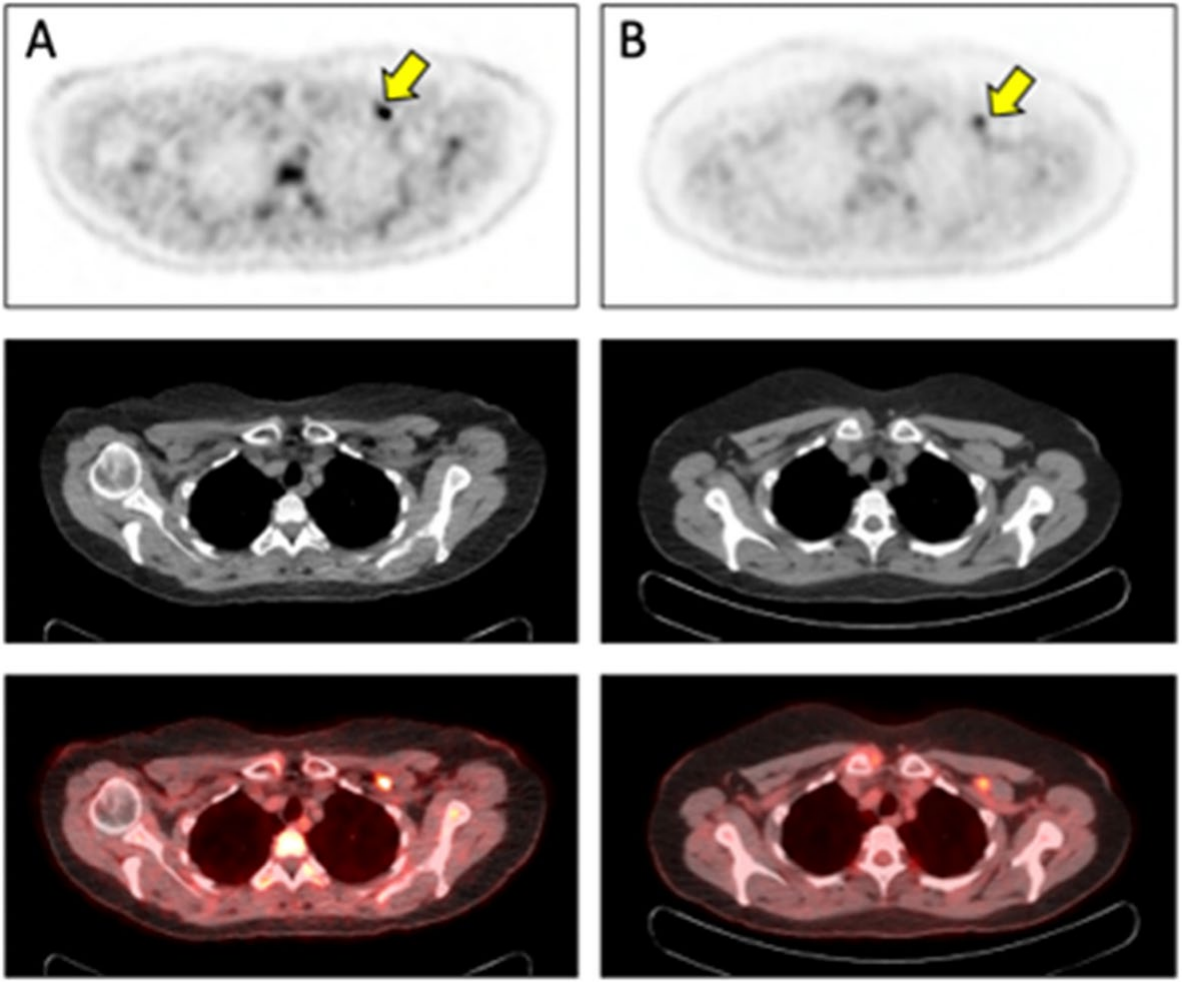
^18^F-Gln-PET/CT (A imaged 67 min post injection) and FDG-PET/CT (B obtained 58 min post injection) images of a patient with ER-expressing breast cancer reveals radiotracer uptake in a left level III lymph node which at pathology demonstrated metastatic ER-positive invasive lobular carcinoma. ^18^F-Gln SUV_MAX_ = 6.4 and FDG SUV_MAX_ = 3.9. High uptake of ^18^F-Gln is reflective of large cellular glutamine pool size and low glutamine catabolism, expected for ER-positive tumors [151]. Modest FDG uptake is also consistent with the modest level of glucose metabolism that is typical for lower grader ER-positive tumors

Glutamine itself has also been radiolabeled to image glutaminolysis, a pathway often up-regulated in malignancy, most notably secondary to the MYC oncogene [148]. ^11^C-glutamine has been developed and studied in animals [149] and holds promise as a research tool, but rapid metabolism and a short half-life preclude widespread clinical adoption. A fluorinated agent, ^18^F-(2S, 4R)4-Fluoroglutamine (^18^F-Gln), has also been developed and advanced into clinical trials. Several malignancies have been imaged in patients, including several breast cancer subtypes [150]. This tracer utilizes the same transporters as native glutamine, but is only minimally metabolized. As such, ^18^F-Gln measures glutamine pool size, an indirect measure of tumor glutaminolysis [150, 151]. In mouse models of breast cancer, TNBC xenografts demonstrated relatively low uptake of ^18^F-Gln, indicative of a small pool size of glutamine in a tumor with inherently high glutamine metabolism. In ER-positive tumors, increased uptake of ^18^F-Gln was observed, reflecting an increased glutamine pool size in this tumor subtype that does not actively consume glutamine. This may also explain good uptake of compounds that reflect glutamine transport and pool in ER-positive tumors (see preliminary result in Fig. 3), including ^18^F-fluciclovine [145]. In the pre-clinical study, an inhibitor of the enzyme that converts glutamine to glutamate was administered, and was shown to increase ^18^F-Gln uptake in TNBC xenografts, reflecting an increase in glutamine pool size as a result of inhibition of downstream inhibition. This suggests a biomarker role for ^18^F-Gln in guiding targeted glutamine therapy [150].

### Other targets and imaging agents

Poly (ADP-ribose) polymerase-1 (PARP-1) inhibitors (PARPi) leverage deficiencies associated with homologous repair that are conferred by tumor mutations in BRCA-related genes, and have proven efficacy in breast cancer. However, response to PARPi is variable despite selection for patients based on known genetic mutations [152]. Imaging PARP-1 expression with the novel PET radiotracer ^18^F-FluorThanatrace (FTT) has also advanced into clinical trials. Imaging PARP inhibitor drug target expression with FTT may enable better patient selection. Variable FTT uptake was seen among patients with and without BRCA mutations, supporting continued study with this tracer as a biomarker [153]. In addition, prostate specific membrane antigen (PSMA) PET, currently approved for imaging tumor cells in prostate cancer, has been also been studied in breast cancer as a marker of neovascularity, given PSMA expression in endothelial cells [154]. An ongoing trial studying PSMA-PET in breast cancer as a biomarker of androgen resistance in HER2-negative, AR-positive breast cancer is ongoing (NCT04573231), noting such an association in prostate cancer. Finally, while not yet well-studied in breast cancer, methods to image immunotherapy targets and immune activation [155, 156] may help guide the rapidly emerging use of immunotherapy in breast cancer.

## Conclusion

The ongoing development and utilization of novel targeted breast cancer drug therapies has transformed clinical breast oncology and improved prognoses. With these new therapies targeting specific biologic pathways comes great potential value for molecular imaging. Molecular imaging offers a noninvasive bioassay to predict and assess early response to treatment across the entire disease burden. This review highlights the most promising PET breast cancer biomarkers to date for early response assessment and for predicting long-term response, including tracers that are currently approved for clinical use, as well as several that are still exploratory, but warrant further study in larger clinical trials.

FDG remains the most widely utilized molecular imaging agent in clinical oncology, including for breast cancer. While FDG-PET/CT has an established role in clinical oncology for staging and restaging advanced breast cancer, its potential applications continue to expand. Research studies have clearly demonstrated the value of FDG-PET/CT for providing an early assessment to neoadjuvant therapy [26, 27], including HER2-targeted therapy [42]. Similarly, studies to date have shown that FDG-PET/CT can provide an early prediction of treatment response in the setting of metastatic, including bone-predominant, breast cancer. However, FDG-PET/CT remains underutilized in clinical oncology for these expanded applications. FDG-PET/CT could have a greater role in the early assessment of neoadjuvant response if future prospective trials clearly define and test adaptive strategies for changing or adjusting neoadjuvant therapy regimens based on early- or mid-therapy PET results [18].

Although FDG is the most established PET tracer in clinical breast oncology, research suggests potential value of multiple novel PET tracers. FES-PET/CT is already approved for defined indications, but research suggests a greater potential role, including for predicting and assessing response to endocrine therapy. Early studies also show promise for FLT, HER2-imaging tracers, and amino acid metabolism tracers. At this point, larger studies, including multicenter trials, are needed to better define appropriate clinical applications, and to provide the foundation for clinical translation. In addition, for some of these newer tracers, such as FLT, prospective studies need to be conducted in more homogeneous tumor populations (ie single subtypes of breast cancer) and in patients on defined treatment regimens, in order to clarify conflicting results that occur among more heterogeneous studies. Finally, standardized methods for PET imaging acquisition and analysis is needed for novel tracers in order to optimize methods for both research and clinical translation. Multiple PET tracers are poised to make an impact in clinical oncology, pending these next steps in investigation.

## Data Availability

Not applicable.

## Abbreviations

PET: Positron emission tomography
FDG: 2-^18^fluoro-2-deoxy-D-glucose
CT: Computed tomography
IHC: Immunohistochemistry
ER: Estrogen receptor
SUV: Standardized uptake value
HER2: Human epidermal growth factor 2
TNBC: Triple negative breast cancer
PgR: Progesterone receptor
SUV_max_: Maximum standardized uptake value
PCR: Pathologic complete response
PPV: Positive predictive value
NPV: Negative predictive value
SUL_max_: Maximum standardized uptake value corrected for lean body mass
NCCN: National Comprehensive Cancer Network
FES: 16α-^18^F-fluoro-17β-estradiol
FFNP: 21-^18^F-fl uoro-16α,17α -[(R)-(1′ -α -furylmethylidene)dioxy]-19-norpregn-4-ene-3,20-dione
AR: Androgen receptor
EGF: Epidermal growth factor
SPECT: Single photon emission computed tomography
ADAPTS: Albumin-binding domain derived affinity proteins
FBEM: N-[2-(4-^18^F-fluorobenzamido)ethyl]maleimide
FLT: ^18^F-3′-deoxy-3′-fluorothymidine
TK-1: Thymidine-kinase-1
CDK 4/6: Cyclin-dependent kinase 4/6
^18^F-fluciclovine: Anti-1-amino-3-^18^F-flurocyclobutane-1-carboxylic acid
ILC: Invasive lobular carcinoma
IDC: Invasive ductal carcinoma
^18^F-Gln: ^18^F-(2S, 4R)4-Fluoroglutamine
PARP1: Poly (ADP-ribose) polymerase-1
PARPi: PARP-1 inhibitors
FTT: ^18^F-FluorThanatrace
PSMA: Prostate specific membrane antigen
